# *Ex vivo* Manufactured Neutrophils for Treatment of Neutropenia—A Process Economic Evaluation

**DOI:** 10.3389/fmed.2019.00021

**Published:** 2019-03-01

**Authors:** Mario A. Torres-Acosta, Richard P. Harrison, Elizabeth Csaszar, Marco Rito-Palomares, Marion E. G. Brunck

**Affiliations:** ^1^Centro de Biotecnología FEMSA, Tecnológico de Monterrey, Monterrey, Mexico; ^2^Centre for Biological Engineering, Holywell Park, Loughborough University, Loughborough, United Kingdom; ^3^Wolfson Centre for Stem Cells, Tissue Engineering and Modelling (STEM), School of Medicine, Nottingham, United Kingdom; ^4^Centre for Commercialization of Regenerative Medicine, Toronto, ON, Canada; ^5^Escuela de Medicina y Ciencias de la Salud, Tecnológico de Monterrey, Monterrey, Mexico

**Keywords:** bioprocess modeling, neutrophils, granulocytes transfusion, acute myeloid leukemia, economic analysis, cost of goods

## Abstract

Neutropenia is a common side-effect of acute myeloid leukemia (AML) chemotherapy characterized by a critical drop in neutrophil blood concentration. Neutropenic patients are prone to infections, experience poorer clinical outcomes, and require expensive medical care. Although transfusions of donor neutrophils are a logical solution to neutropenia, this approach has not gained clinical traction, primarily due to challenges associated with obtaining sufficiently large numbers of neutrophils from donors whilst logistically managing their extremely short shelf-life. A protocol has been developed that produces clinical-scale quantities of neutrophils from hematopoietic stem and progenitor cells (HSPC) in 10 L single-use bioreactors (1). This strategy could be used to mass produce neutrophils and generate sufficient cell numbers to allow decisive clinical trials of neutrophil transfusion. We present a bioprocess model for neutrophil production at relevant clinical-scale. We evaluated two production scenarios, and the impact on cost of goods (COG) of multiple model parameters including cell yield, materials costs, and process duration. The most significant contributors to cost were consumables and raw materials, including the cost of procuring HSPC-containing umbilical cord blood. The model indicates that the most cost-efficient culture volume (batch size) is ~100 L in a single bioreactor. This study serves as a framework for decision-making and optimization strategies when contemplating the production of clinical quantities of cells for allogeneic therapy.

## Introduction

Shortages in the availability of blood and blood components are a persistent challenge worldwide (2–5). This includes commonly used components such as universal donor erythrocytes (red blood cells, RBC), and platelet concentrates used in medical emergencies. Neutrophils, the most abundant type of leukocyte in human blood, are also of interest for transfusion therapy in niche applications (6). However, multiple constraints in donor cell collection and processing currently make neutrophil transfusion unrealistic on a routine basis (7).

To meet this increasing demand for blood cells, researchers have pursued *in vitro* production, with the overarching goal to generate a limitless supply of safe and potent cells for transfusion. Hematopoietic stem and progenitor cells (HSPC) that give rise to all lineages of blood cells can now be generated from somatic (8) and pluripotent stem cells (9) in the laboratory. Research protocols can yield large-scale numbers of platelet-producing megakaryocytes (10), erythrocytes (11, 12), and neutrophils (1). Similar to donor blood transfusions, these *in vitro* produced blood cells are targeted toward allogeneic transfusions. In addition to solving supply issues, generating blood cells *in vitro* would allow standardization of blood product composition, which in turn eliminates the risks of infectious disease transmission (13), and graft vs. host disease (GvHD) (14, 15). It may also provide an opportunity to develop superior products, for example to address alloimmunization complications in patients who need recurrent transfusions (16, 17).

High cost of goods (COG) is a major cause of commercial failure of cell therapies (18). To avoid this pitfall, considering cost of production early in development is critical. We wished to investigate the bioprocess and associated costs in the production of blood components at clinical-scale. While economic analysis on production bioprocesses for allogeneic mesenchymal stem cell (MSC) therapies are available (19–22), major differences in the bioprocesses make these studies inadequate to evaluate COG for the production of blood cells *in vitro*. Unlike blood cells, MSC are anchorage dependent, driving specific approaches to cultivation and downstream processing (21). In addition, the number of cells required in MSC therapy may be significantly lower than those required for clinical efficacy of blood components (in the range of 10^3^-10^6^ for MSC vs. 10^10^-10^12^ for neutrophils and erythrocytes, per respective dose), leading to very different process considerations (22). Bioprocesses for blood cell production are also very different from other mammalian cell culture processes such as for hybridomas or CHO cells used commercially to produce monoclonal antibodies. These cell types can withstand comparatively harsh culture conditions, and as the cells themselves are not collected, maintenance of cell health and function in not a strict process requirement.

Here we propose a bioprocess model and present an economic analysis for the production of *in vitro* generated neutrophils (iNeut) at clinically significant scale, as a case study for *in vitro* production of blood components. This study will serve as a framework for decision-making when contemplating the production of clinical quantities of iNeut. Furthermore, it will form the basis for optimizing production strategies utilizing COG as a key metric. We anticipate these results will be applicable to a range of *in vitro* produced allogeneic cell therapies with inherently challenging production, storage and logistical requirements.

## Case Study

Patients undergoing chemotherapy for hematological malignancies often experience a neutropenic period that dramatically increases the risk of infection, despite the use of prophylactic antibiotics and antifungals (23, 24). In these patients, replenishing the pool of neutrophils through transfusions until recovery of the endogenous population seems logical. However, intrinsic attributes of donor neutrophil products, such as contaminants, short half-life and complicated collection processes, have hampered adequate clinical trials and precludes their use as common practice. As an alternative to donor neutrophils, iNeut can be produced in the laboratory at clinical-scale in a bioreactor, using CD34^+^ HSPC enriched from umbilical cord blood (UCB) as starting material (1, 25). Using such approaches, iNeut could be produced in advance of clinical need in large batches, cryopreserved and tested, and made available for clinical use as a safe and consistent off-the-shelf cell product.

Prophylactic transfusions of iNeut may be preferred to treatment of a pre-existing infection, as less cells are required to achieve protection compared to clearing an infection. The success of prophylactic neutrophil transfusions is described in several studies (26, 27). Furthermore, waiting for signs of infection may select for patients with infections too advanced to allow recovery (7, 28). The number of cells required in a protective dose is estimated at ≥ 2 × 10^10^ iNeut given every second day (1, 26) for the duration of neutropenia. In an acute myeloid leukemia (AML) setting where neutropenia is a frequent complication, the duration of neutropenia is between 7 (29) and 29 days (30).

The American Cancer Society projects >21,000 new cases of AML in the United States in 2017 (31). An estimated 50–90% of these patients will experience neutropenic infections (23, 24), which equates to ~18,900 patients. Assuming successful clinical trials of prophylactic iNeut, it is reasonable to propose 10% market penetration (1,890 patients) of the iNeut product for the entire AML population each year, with a single neutropenic episode per patient. The average duration of neutropenia is 2 weeks, calling for 7 transfusions of neutrophils as a bridge treatment for each neutropenic episode (totaling 13,230 doses per year).

Assuming a 335 day-per-year working facility, a 15-day manufacturing process can produce 22 consecutive iNeut batches yearly. Therefore, a single wave-mixed bioreactor such as the Wave Bioreactor™ 500/1,000 (GE Healthcare) could produce 1,100 doses per year [one dose of 2 × 10^10^ iNeut per 10 L, (1)], and 12 such bioreactors would be required to meet demand. This estimate led us to focus this study on the bioprocess and its associated costs in the production of iNeut in a single bioreactor. Our initial aim is to provide probable costs for clinical-scale manufacture to support clinical trials. By analyzing key cost drivers, we identify areas for optimization to reach cost effective production while maximizing cellular output.

This modeling exercise evidenced the current most cost-efficient protocol to produce allogeneic neutrophils *ex vivo* for transfusion (27-day culture to yield 104L culture per bioreactor). Depending on the available discount for bulk purchase of consumables, and the cost of UCB purchasing, the CoG for a single prophylactic dose ranges between US$1,607-US$7,448. A full treatment, assuming a 7-day neutropenic window reported in AML patients, would require 3 transfusions, with full treatment CoG for production ranging between US$4,821-US$22,344. This cost represents a fraction of what the cost of administering the therapy would be, as several relevant parameters are not included in this work, such as transport and infusion costs.

In the UK, the cost of primary prophylaxis with Filgrastim was calculated as £12,147 for 6 days in a cohort of breast cancer patients in 2011 (32). Adjusting for inflation and currency at the time of writing, this is equivalent to US$19,165. Another study reports an average cost of US$1,928 (adjusted for inflation and currency) for the use of Filgrastim during induction chemotherapy in AML patients. Of note, this cost only includes the purchase of the drug. Interestingly, Filgrastim during induction chemotherapy did not improve outcomes such as incidence of fever or hospitalization, nor median duration of neutropenia during induction chemotherapy, suggesting Filgrastim treatment may not be an ideal option in these patients (33). We suggest further studies are necessary to identify patients Willingness-to-Pay, manufacturing protocol optimizations to reduce costs, as well as efficacy of the treatment *in vivo*, and possible synergy of G-CSF infusions and *ex vivo* manufactured neutrophil transfusions, to benefit patients.

## Materials and Methods

### Rationale for Scale Selection and Study Design

We wished to evaluate manufacturing costs of neutrophils produced in a closed system at clinically relevant scales, in a single bioreactor, using Biosolve (Biopharm Services Inc., Buckinghamshire, UK) for bioprocess modeling. CD34^+^ HSPC can be expanded 5,800-fold, and differentiated into neutrophils over 15 days in a wave-mixed bioreactor (1). The bioprocess model considered batch volumes that span the current capacities of commercially available Wave Bioreactors using disposable technology: from 12.5 to 500 L working volume, equivalent to 2.5 × 10^10^ to 1 × 10^12^ cells, or 1 to 50 iNeut doses (with 1 dose ≥ 2 × 10^10^ cells) (1, 26). Further increases in manufacturing capacity could be achieved via scale-out approach using multiple bioreactors (and a multiplication of batch production costs). While other bioreactor platforms (e.g., stirred-tanks) might also be suitable for iNeut culture, we focus on the already demonstrated use of wave-mixed bioreactors.

Equipment size constraint in scaling up production within the 12.5 and 500 L working volume range, only applied to the bioreactor in this bioprocess. This is because HSPC enrichment can be performed upstream in multiple batches and frozen until initiation of culture, while downstream processing using fluidized bed centrifugation (FBC) with for example the kSep®400 system (Sartorius group)^1^ can be achieved over several batches without affecting initiation of a new batch. However, there is no commercially available single Wave Bioreactor that can sustain the full spectrum of culture volumes we propose to analyze. The ReadyToProcess Wave 25 and Xuri™ Cell Expansion System W25 both have a working capacity ranging from 0.1 to 12.5 L. The Wave Bioreactor System 200 has a culture volume range of 10 to 100 L, and finally the largest commercially available Wave Bioreactor System 500/1,000 can sustain culture volumes between 50 and 500 L (all, GE Healthcare Life Sciences). Overlapping capacity between bioreactors created a bottleneck in equipment selection when 2 bioreactors could support the production scale selected. For this reason, we created an equation, using the Biosolve database of costs, to predict equipment and consumables costs depending on culture volume, regardless of bioreactor possible upgrade. As a result, although no commercially available bioreactors can fit all possible scales analyzed, a theoretical Wave Bioreactor equipment cost was computed for culture volumes between 12.5 and 500 L using a 0.5 L increment for each new scale, to generate continuous and consistent data (Supplementary Table 1).

### Model Set-Up in Biosolve

**Figure 1:**
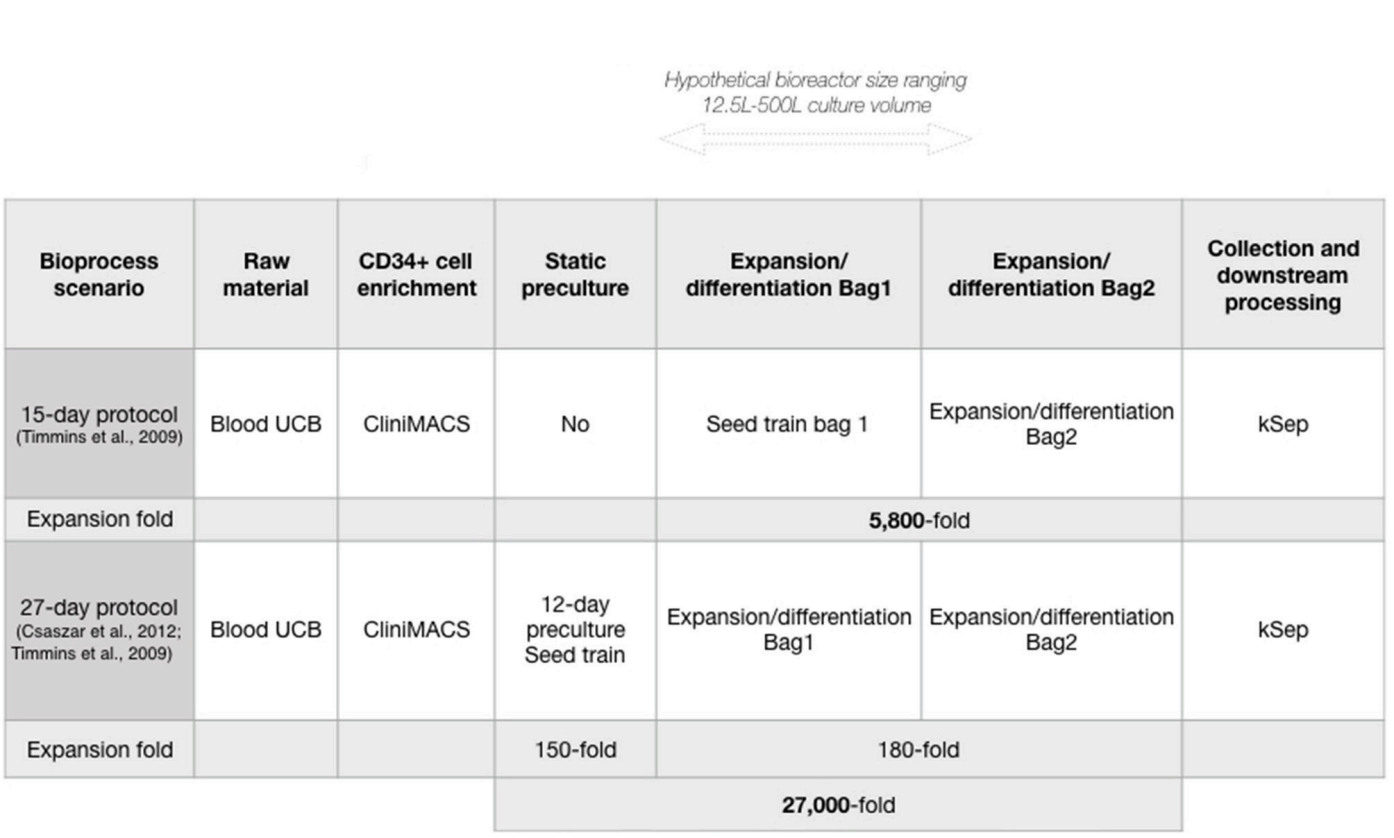
Production bioprocess scenarios to generate neutrophils *in vitro* at large-scales.

The Biosolve model was initially populated using published data of the production of neutrophils at bioreactor-level (1), starting with UCB-derived CD34^+^ HSPC. Timmins et al. showed a linear correlation between cell yield and culture volume, regardless of the culture protocol used (static flasks or disposable equipment in wave-mixed bioreactor) over a 15-day culture. Consequently, we have made the assumption that production data at the experimentally validated 10 L culture volume can be used to predict cell yield in further scale-up in similar bioreactor technologies. As this process is focused on developing a cell therapy product from HSPC, the sequence of unit operations contains a purification step to collect CD34^+^ HSPC from UCB (CliniMACS® system, Miltenyi Biotec), followed by a cell expansion/differentiation step (Wave Bioreactor, GE Healthcare Life Sciences), and finally a cell harvest/downstream processing step using FBC (kSep system, Sartorius), all occurring in enclosed, disposable systems (Figure 1A). In this bioprocess, FBC was selected over its tangential flow filtration alternative, which may cause increased cell loss, and cell activation (34, 35). In order to propose a full model, four distinct datasets must be fed: 1-capital, 2-labor, 3-materials, and 4-consumables (cost data detailed in Supplementary Table 1).

Capital (reflected as “equipment contribution”) was the sum of costs of major equipment as no facility building was considered. To determine the capital contribution to production cost (per batch or per 10^8^ cells), we used a 12% interest rate over a 10-year period. Yearly loan repayment was divided by the number of batches performed per year (depending on the analyzed production scenario) and then by the number of produced cells to obtain the annual charge per cell yield.

Labor was adjusted to reflect 15% of production costs on the base scenario of 12.5 L working volume (36). The obtained absolute value was set constant at greater production scales as the complexity of the process remains unchanged throughout scale-up (i.e.,: a single Wave Bioreactor would be operated).

Materials comprise the basal chemically-defined culture media (Stemline® II, Sigma-Aldrich) and the expansion/differentiation cytokines cocktail: Stem Cell Factor (SCF, Sigma-Aldrich), Granulocyte-Colony Stimulating Factor (G-CSF, Sigma-Aldrich) and Thrombopoietin Peptide Mimetic (TPO_pep_, CanPeptide) used for cell culture. Possible economies of scale are speculative, therefore we envisaged two scenarios of either 30 or 90% discount on prices reported by suppliers for laboratory scale experiments.

Consumables are disposables involved in the bioprocess such as single-use culture bags and continuous flow centrifugation consumables used for downstream processing. The feeding strategy developed for this process (1) requires two culture bags per batch: a bag of final production volume capacity and a bag of capacity one-tenth of the final volume size (for seed train). Culture bags must be operated at a maximum of half their full capacity, by recommendation of the supplier (GE Healthcare Life Sciences). Therefore, despite factoring in the largest commercially available wave bioreactor supporting a 1,000 L disposable bag, the maximum culture volume analyzed was 500L. All volumes mentioned below are actual culture volumes. Quality control costs were estimated equal to labor costs (36).

A critical contributor to this model is the expense generated by sourcing the CD34^+^ cells from UCB. Umbilical cords banked for cell therapy purposes can be accessed at a range of highly varying prices, depending on location and material quality (CD34^+^ cell content). In Canada/United States access costs range from US$2,500 to US$30,000 depending on cord quality (from 2 to 5 × 10^6^ cells/UCB for low to high quality cords, respectively) [personal communication Dr. Elizabeth Csaszar, and (37)]. In other geographical locations, acquisition costs vary significantly, to only cover transportation costs recovery (~US$500 in Mexico) (38). This study considers no UCB costs, low cost (US$ 2,500) and high cost (US$ 30,000).

This work presents costs classified as “Equipment Contribution,” “Materials,” “Consumables,” “Labor,” “UBC Cost,” and “Other.” This was done in order to capture within a single number the overall production cost of a batch, which in turn depends on the scale being analyzed. As traditional cost classification contains fixed and variables costs, or investment and production costs, we implemented the following distribution to simulate a conventional classification: fixed costs will include equipment contribution and labor, while variable costs will be materials, consumables and the UBC acquisition cost. Investment costs consider the overall cost of acquiring equipment only (noting that no production plant was designed), with the remaining costs classified as production costs.

A deterministic analysis was performed to understand variations in production costs per batch (COG/batch) and per 10^8^ cells produced (COG/10^8^ cells). Each possible single batch volume was evaluated (as a continuum between 12.5 and 500 L working volume), and its respective production cost was registered together with its composition to identify costs drivers at each scale. Biosolve breaks down the production cost by materials, consumables, labor and “other” (waste disposal, maintenance and insurance). A program was written in Visual Basic (Microsoft Office 365 Pro Plus, Microsoft Corporation) to generate and input each possible production volume into Biosolve, which calculated cost composition for each scenario. The program adjusted volumes of consumables and materials required.

### Bioprocess Scenarios

We next used the developed model to analyze an alternative production scenario, where 12 days of pre-expansion culture of CD34^+^ cells increased CD34^+^ cell numbers before initiating neutrophil differentiation (39). Briefly, a conditioning media containing SCF, thrombopoietin (TPO) and FMS-like Trysine Kinase 3 Ligand (FL) is delivered semicontinuously to HSPC culture to maintain a cell concentration that promotes enhanced expansion in small volumes. This protocol was used in combination with the 15-day iNeut expansion/differentiation protocol, to create a novel bioprocess of 27 days that generates an overall 5.4-fold increase in cell number over the 15-day original process (Figure 1).

To analyze costs involved in the 27-day bioprocess, we maintained all parameter values identical to the matched scale using the 15-day process, expect for batch duration (12 day-increase) and labor (adjusted to reflect additional labor brought on by the extra 12 days). Increased use of materials was insignificant in the overall production cost due to the very low culture volume over the first 12 culture days.

### Sensitivity Analysis

Variations in model parameters can have a dramatic impact on production cost (36). Such parameters encompass production yield (total cell production), downstream processing recovery yield, costs of materials/consumables and process duration (40). A sensitivity analysis was performed to evaluate potential parameter impacts. Parameter values were individually modified and resulting production costs fluctuations were recorded (COG/batch and COG/10^8^ cells). To modify parameter values, best and worst scenarios were created for production yield, batch duration and consumable costs (Table 1). Culture yield and duration variations were based on the reported standard variations (plus and minus 1 value of standard deviation) for iNeut production in the original study (1). For materials/consumables costs scenarios, we used ± 25% (41) of the 90% discounted costs. A sensitivity analysis was performed for both production protocols (15- and 27-day), using the range of culture volume mentioned in the previous section.

**Table 1:** Production scenarios designed for the sensitivity analysis.

	**Production scenarios**	
	**Worst**	**Best**
Cell yield (%)	−30	+30
Materials costs variation (%)	+25	−25
Batch duration (days)	+3	−3

*This analysis was performed for the 15 and 27 day protocols, adjusting accordingly to capture the variations as described*.

## Results and Discussion

### Deterministic Analysis

#### Production Scenarios

Here we study the behavior of costs associated with producing neutrophils *in vitro* in a single-use bioreactor, to identify economic bottlenecks in small settings productions, such as for a clinical trial. This study also informs on likely treatment production costs for small commercial scale.

A major bottleneck in cell therapy bioprocesses is the generation of commercially and clinically relevant number of cells. In an effort to improve cell output, a 27-day protocol was developed using preliminary expansion of the HSPC at the front-end of the 15-day protocol, as a seed train (Figure 1). While it almost doubles batch duration, the 27-day protocol generates an overall 27,000-fold expansion, corresponding to a 5.4-fold improvement over the 15-day protocol (Figure 1). Comparing both protocols was warranted to investigate economic advantages of the 27-day protocol. Both production scenarios (15 and 27-day protocols) similarly affected the overall trend of COG despite different absolute numbers.

**Figure 2:**
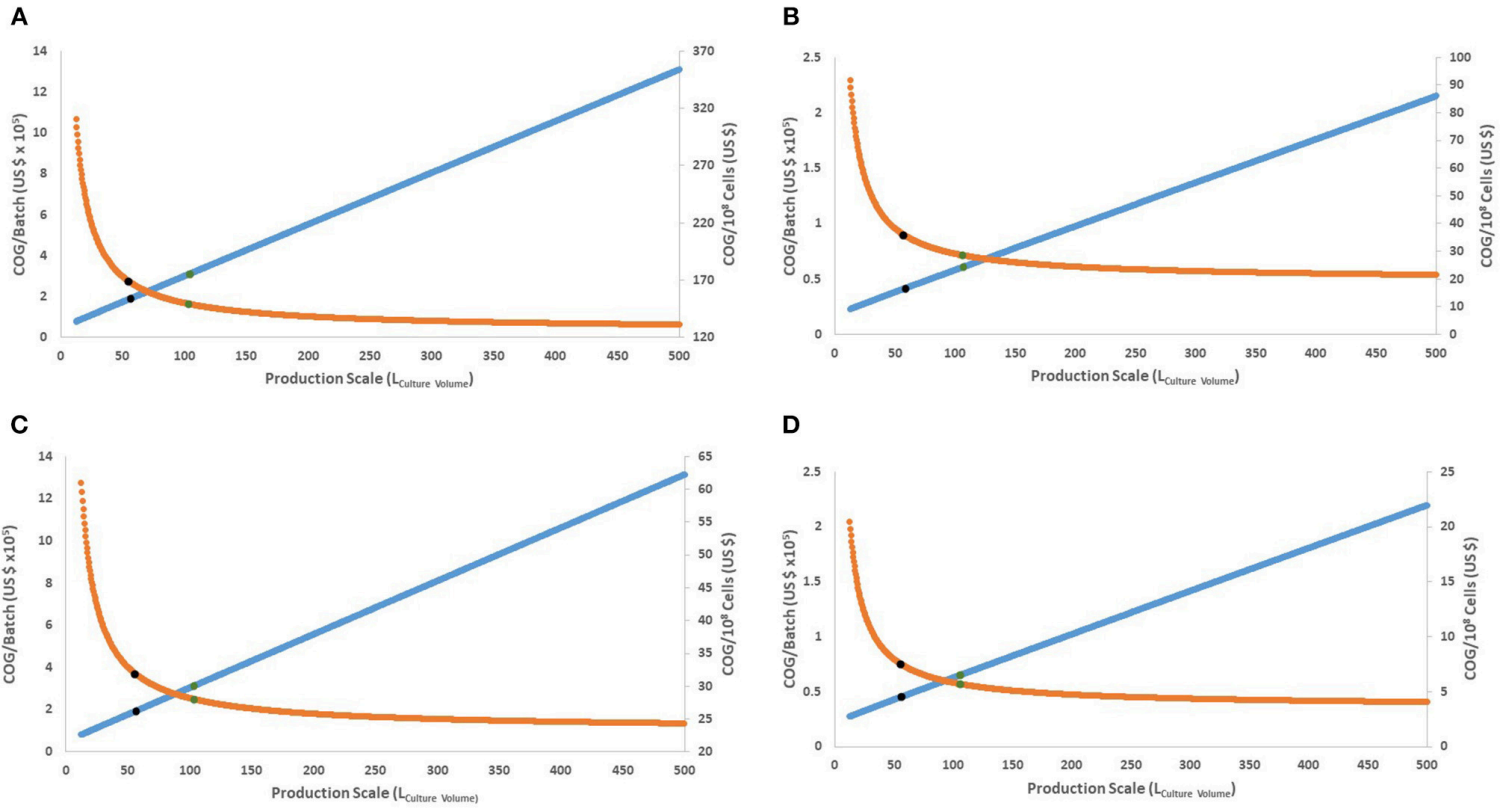
Behavior of COG/10^8^ cells and COG/batch for the full range of culture volumes possible in a Wave Bioreactor (12.5–500 L of culture volume). **(A,B)** present the 15-day protocol with 30 or 90% discount for bulk materials, respectively. **(C,D)** present the 27-day protocol with 30 or 90% discount for bulk materials, respectively. Orange line represents COG/10^8^ cells. Blue line represents COG/batch (× 10^5^). Blue dots represent the culture volume with 80% improvement, while green dots for 90%.

Scaling-up production of iNeut using the 15-day or 27-day protocols linearly increased the COG/batch (Figure 2). This is explained by the requirement for larger equipment, and incremental intensification in the use of materials and consumables. In reality, equipment size is only modified upon incrementing batch volume if the analyzed scale surpasses the capacity of the bioreactor used for the previous scale analysis. However, within the Wave Bioreactor range some volumes can be sustained by two reactors, for instance a 90 L culture volume may be produced using either the 200 or 500/1,000 systems. Including an analysis of volume optimization in these situations was beyond the scope of this manuscript. We aimed to predict equipment and consumable cost depending on culture volume, regardless of possible bioreactor upgrade. Therefore, we decided to consider a theoretical [Wave Bioreactor + disposable bag] cost that is sequentially incremented together with the selected batch size to be analyzed (Supplementary Table 1).

As production costs increase, successively larger batches yield more cells which in turn decreases the COG/10^8^ cells. As production scale increases, COG/10^8^ cells follow a logarithmic asymptotic curve (Figure 2). This creates a theoretical limit to expanding the bioprocess size, where further increment will not significantly favor COG/10^8^ cells. Moreover, if the potential decrease in COG/10^8^ cells is calculated as per Equation (1),

(1) $$\begin{array}{r} \frac{\frac{COG}{10^{8}\;cells_{Given\;Production\;Scale}}-\;\frac{COG}{10^{8}\;cells_{500\;L}}}{\frac{COG}{10^{8}\;cells_{12.5\;L}}-\frac{COG}{10^{8}\;cells_{500\;L}}}\;\times \;100\% \end{array}$$

Equation (1): Theoretical cost improvement (%) based on lower and upper limit culture volumes in a Wave Bioreactor.

Both protocols have similar culture volumes favoring cost improvement, regardless of material costs (Supplementary Table 2). Eighty % of maximum possible decrease in COG/10^8^ cells is achieved by using ~58 L culture volume and 90% is achieved using ~105 L culture volume (Figure 2). This translates into a rational 105 L production volume upper limit per bioreactor (210 L full size hypothetical bioreactor), equivalent to 21 × 10^10^ cells (or 10 doses) per batch.

The most cost-efficient scenario (90% possible optimization) was using the 27-day protocol at the 105 L scale (Figure 2). Assuming a conservative 30% bulk discount of material costs, the 27-day protocol yielded an approximate saving of US$ 24,217 per dose (2 × 10^10^ iNeut) over its 15-day counterpart (or a saving of US$ 121 per 10^8^ cells) (Figures 2A,C), disregarding UCB purchase costs which are discussed below.

#### Cost of UCB Acquisition

**Figure 3:**
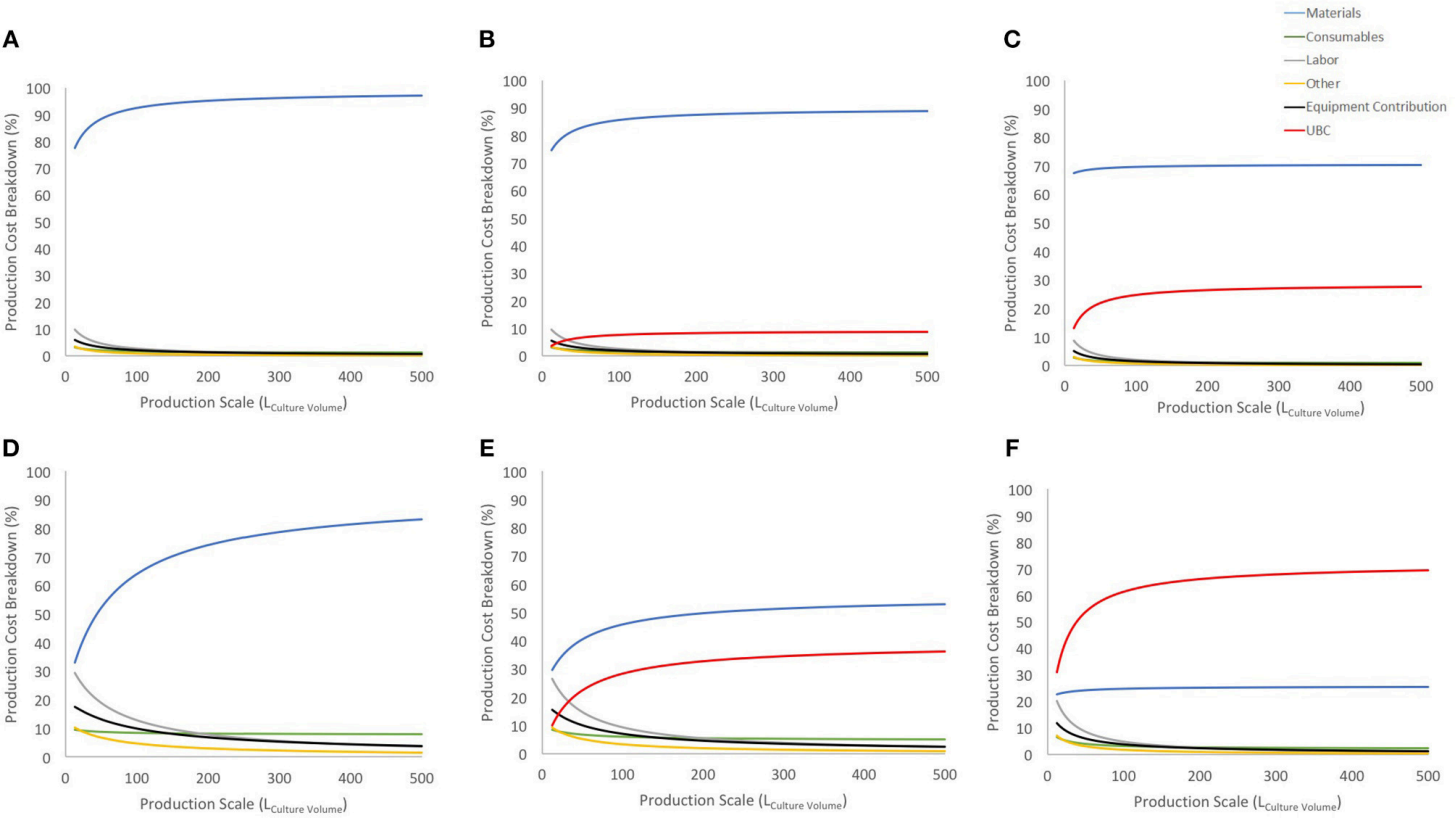
Cost breakdown for the COG/10^8^ with and without UCB costs (US$ 2,500 and US$ 30,000). **(A–C)** show breakdown for the 27-day protocol with a 30% discount on materials bulk price for no cord cost, US$ 2,500 cord and US$ 30,000 cord, respectively. **(D–F)** shows the same information and order for 90% discount on materials bulk price. Blue line shows materials contribution, consumables in green, labor in gray, other in yellow, equipment contribution in black and UCB cost in red.

Once the 27-day production scenario was evidenced as most cost efficient, production costs (COG/10^8^ cells) were dissected for each scale to analyze their breakdown (Figure 3). A significant COG component was acquisition cost of CD34^+^ HSPC-containing UCB. Although UCB is a readily available, non-invasively collected source of HSPC used in cell therapy post *in vitro*-manipulation (42), its cost of acquisition may be prohibitive. Due to the great discrepancy that currently exists in UCB acquisition charges; we decided to inform a range of possible costs: higher (US$ 30,000), lower (US$ 2,500), as well as no cost.

Considering a conservative 30% discount on material purchase (Figures 3A–C), UCB acquisition has a limited contribution to the COG at lower scales and stabilizes before 150 L at either 10% or 25% (for US$ 2,500 or US$ 30,000 UCB, respectively). Using a 90% discount on material purchase evidences a similar trend albeit a much higher impact on COG (up to 30 and 60% of COG for US$ 2,500 or US$ 30,000 UCB, respectively). With this scenario, the US$ 30,000 was the highest cost contributor, and more than doubled the contribution of materials (second highest COG contributor) at large scales (Figure 3F).

Regardless of possible discount on material costs, UCB contributions to COG evidence an area of opportunity to optimize their purchase. While there is in theory 3 times more CD34^+^ cells in a “high quality” UCB, it is 12 times more expensive than its “lower quality” counterpart. Should CD34^+^ content be the only contrast between “lower” and “high” quality UCB, it is therefore more cost-effective to triple purchase “lower quality” UCB.

However, CD34^+^ content may not be an accurate indicator of UCB quality. Recent modifications were made to required quality control (QC) of UCB for transplantation to include potency assays, pre- and post-cryopreservation in addition to concentration and viability (43). Although there is no direct evidence that the number of colony forming cells present in UCB correlate with higher number of neutrophils produced *in vitro*, colony forming unit (CFU) assay is a better predictor of neutrophil engraftment compared to CD34^+^ cell count in allogeneic UCB transplantation (44). Therefore, it may be relevant to investigate an alternative assay for UCB quality, such as CFU assays for selecting “high expansion potency” UCB. Overall this suggests that the COG contributions above may reflect the impact of CD34^+^ cells number rather than UCB quality defined as expansion potential.

Abrogating dependence on UCB would be possible by using an immortalized cell line to produce neutrophils. Using a cell line would also maintain process consistency by limiting variations in starting material. However, the risk of transplanting the transfused cells in patients is less well-understood. Transfused neoplastic neutrophils collected from chronic myelogenous leukemia patients could sustain neutrophil counts over 52 days, which suggested engraftment in the marrow (45). To limit this risk, HL-60, a neutrophilic cell line, was stably transfected with *UL23* to create cell line-derived neutrophils containing a suicide trap (named ATAK cells) (46). Upon exposure to ganciclovir, DNA synthesis is altered leading to cell death. Transfused ATAK cells improved survival of infected neutropenic mice, accumulated in infected organs and persisted over days, despite a unique transfusion (46, 47). Exposure to ganciclovir depleted these cells *in vivo*. However, these cells were not suitable for clinical translation (Dr. Brad Spellberg, personal communication) and discussing the extensive contrast between HL-60 cells and UCB-derived iNeut is beyond the scope of this study. An alternative is to conditionally immortalize CD34^+^ HSPC to further increase expansion. This strategy was successful at producing large numbers of RBC (12), although in this context cell enucleation upon maturation may limits engraftment risks in transfusion recipient. An alternative strategy to sourcing CD34^+^ HSPC from UCB is their generation *in vitro* from induced pluripotent stem cells (iPSC) (9, 48). While this approach may initially be explored for autologous therapies, protocols that yield high quantities of iPSC-derived CD34^+^ cells could be implemented at the front-end of the 27-day protocol, and compared to the presented UCB-dependent protocol.

#### Material and Labor

Material costs are the dominant COG contributor (disregarding the extreme example of 90% bulk discount combined to US$ 30,000 UCB) (Figure 3), constituting 3 to 7 times more than the next higher contributor when a conservative 30% material discount is considered.

**Figure 4:**
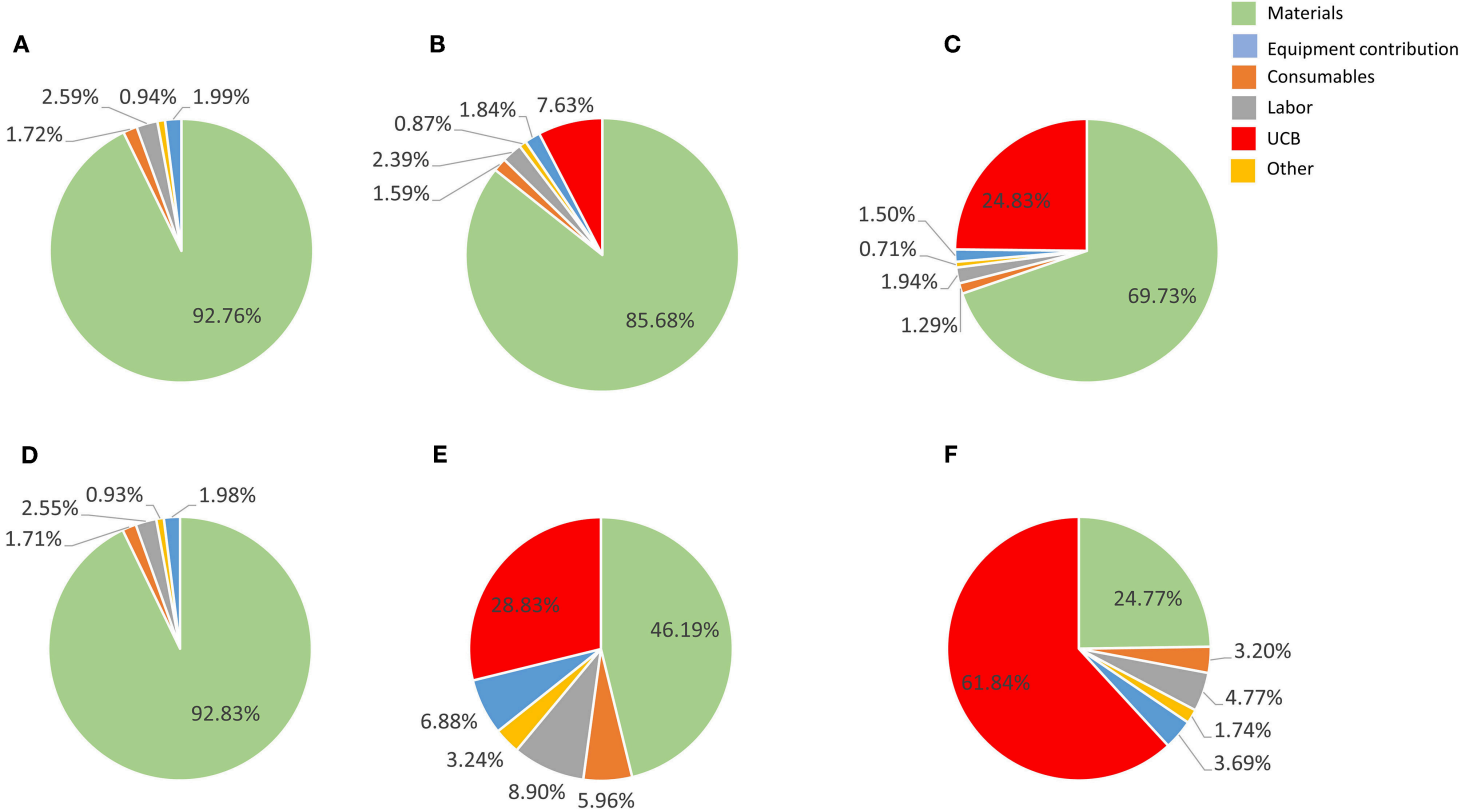
Cost composition for the proposed best scale of operation in a single Wave Bioreactor (90% COG improvement) for the 27-day protocol considering a 30% discount on materials **(A–C)** and 90% discount **(E–F)**. **(A,D)** show results a UCB acquisition cost of zero, **(B,E)** for a US$ 2,500 UBC, and **(C,F)** for US$ 30,000 UCB. Materials are shown in light blue, consumables in orange, labor in gray, other in yellow, equipment contribution in dark blue and umbilical cord cost in green.

Contribution of material costs depend on allocated supplier discount for bulk purchase (which may vary between suppliers) and selection of starting material, as UCB purchase contribution may absorb significantly material contributions (Figures 3, 4). Material costs become more relevant to production COG at large batch size, due to the increased amount of culture media required. At the best production scenario of 104 L over 27 days, material costs contribute between ~between 25 and 93% of COG, hence it is a critical candidate for optimizations.

The culture media used in the studied processes (StemLine II) is defined and complex, including a cocktail of growth factors (SCF, G-CSF and TPO_pep_), and is consequently expensive. A strategy currently explored to decrease material costs is to replace StemLine® with a basal Iscove's Modified Dulbecco's Medium (IMDM) supplemented with minimal additives (Dr. Elizabeth Csaszar, personal communication).

Switching to biosimilar growth-factors is another method to control material costs while maintaining bioprocess efficiency. The media used by Timmins and colleagues already contains a biosimilar in the TPO_pep_ (49). Multiple alternatives exist for G-CSF: Zarzio® (Novartis) may be used in the clinic in place of original pharmaceutical G-CSF such as Neupogen® (Amgen) (50, 51). This substitution may generate >20% saving in cost of patient treatment (52), an economy that may also be applicable to the iNeut bioprocess. Profarma, a Brazilian pharmaceutical company, filed a patent in 2015 for ancestim (SCF) biosimilars (53), suggesting the complete cytokine cocktail used in the iNeut bioprocess may be optimized using cheaper alternatives in the future, although initial investments would be necessary to validate similar efficacy. Recycling growth factors or reducing their consumption may also generated savings on material costs. Magnetically labeled growth factors can be selectively used in closed-system culture (54). This feature may allow for similar expansion while limiting proteins degradation. These examples are non-exhaustive and combining multiple approaches may generate enhanced savings.

Labor is consistently the third highest cost contributor at the best identified production scenario and scale (Figure 4). Human operators contribute significantly not only to increasing the variability in the process, but also the underlying business operating costs. This is particularly true in the current emerging cell therapy industry where there is a chronic shortage of skilled operators. To ameliorate this problem, a number of companies are formulating automated production platforms for manufacturing cell-based therapies, such as the ambr® 250 (Sartorius Stedim formerly TAP Biosystems) which accommodates suspension cultures up to 250 mL (55). Some of these platforms such as the CliniMACS Prodigy® (Miltenyi Biotech) are modular, allowing the unit to be reconfigured for different work-flows (56).

Currently these automated platforms lack many of the features that would enable them to be truly autonomous and require human intervention and supervision regularly. Particularly challenging steps to automate such as initial seed or final transfer steps are likely to remain in the realm of the human operator, but recent advances are bringing more of the online monitoring and routine media exchange steps under automation. These future steps are being catalyzed by advances in reactor design, online monitoring technologies (57) and *novel* separation and purification technologies (58). This progress in automation should present opportunities for reduction on COG alongside increasing homogeneity of manufactured product.

### Impact of Parameter Variations: Bioprocess Sensitivity Analysis

**Figure 5:**
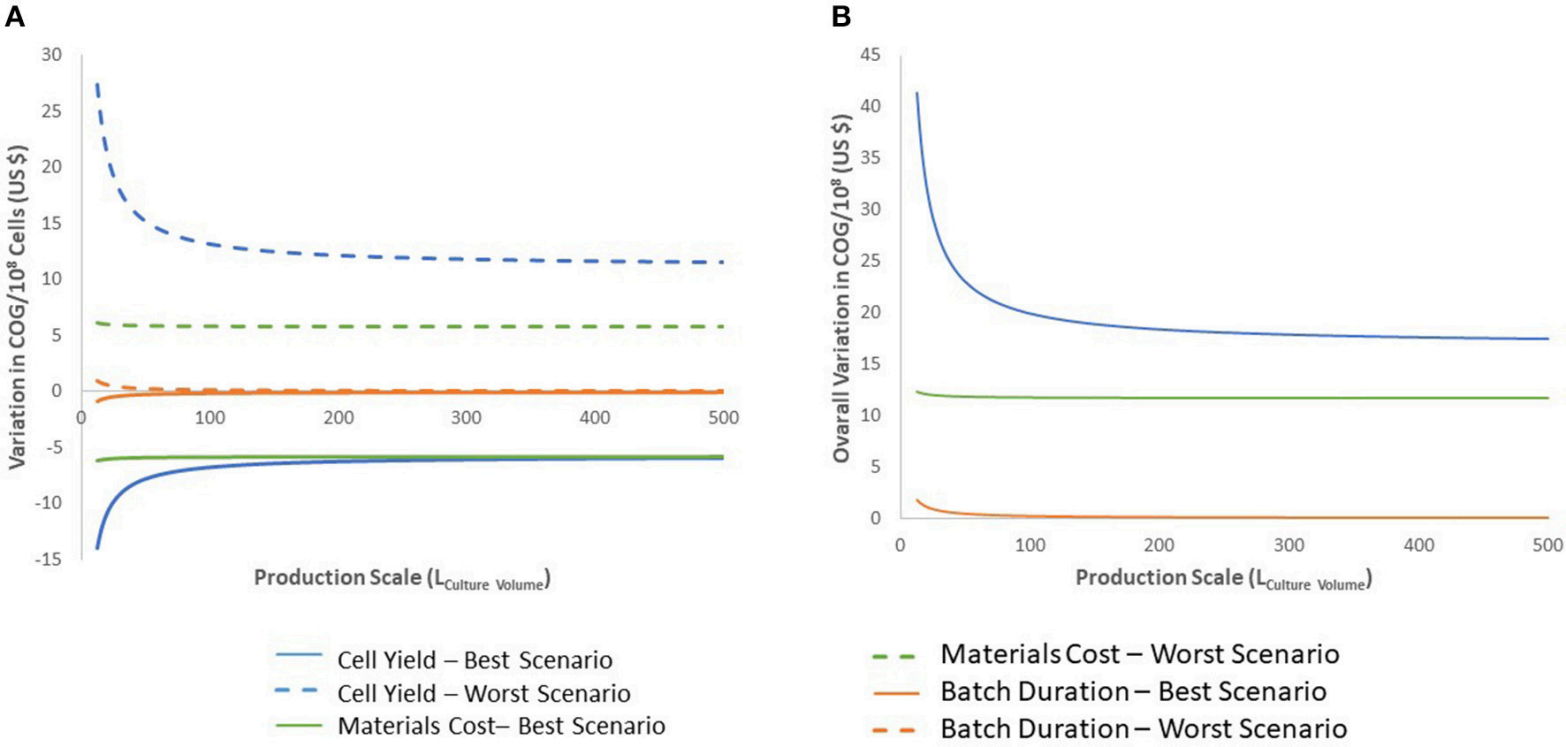
Sensitivity analysis for the 27-day protocol following variation in cell production, materials costs, and batch duration. **(A)** shows the variation of the best and worst scenarios compared with the base calculation, positive values indicate worst scenario results and negative values for best scenario. **(B)** presents the overall variation (complete range from worst to best results). Variables analyzed are indicated in blue for cell production, green for materials costs and orange for batch duration. Dashed lines indicate results for worst scenario and continuous lines for best scenario.

We evaluated the impact of variations in cell yield, batch duration and material costs on COG/10^8^ cells. Final cell yield and variations in material costs markedly impacted COG/10^8^ cells (Figure 5A). Varying final cell yield had the most profound impact on COG, while exhibiting an inflection point around 50 L, it remained the dominant cost driver at all scales. Past 100 L culture, a 30% decrease in cell yield increased COG by almost US$ 15.00 per 10^8^ cells. That is equivalent to a US$ 3,000 COG increase per dose of 2 × 10^10^ iNeut, or US$ 30,000 increase per batch using the best production scale of 104 L.

The impact of material costs variations on COG remained constant at all scales analyzed (Figure 5). Although variations in materials costs are independent from process parameters or a potential optimization, developing processes that sustain higher cell density to produce more cells per batch would optimize material use. This may be challenging as inhibitory feedback signaling is known to impact continued cell expansion at higher densities, a characteristic that was manipulated to further expand HSPC in the 27-day protocol (39).

Batch duration limits the number of batches a facility can operate yearly. Therefore, batch duration impacts yearly cell yield despite a consistent cell yield/batch. From the economic perspective evaluated here, above 50 L culture batches, batch duration does not impact COG (Figure 5). The minimal effect of batch duration on COG/10^8^ cells may be explained by variations in labor. The required quantities of materials and consumables to produce a single batch is fixed. However, longer batches translate to increased labor. Therefore, batch duration affects requirement for staff, but does not influence the materials, consumables or equipment size needed in the production of the batch. In consequence, optimization efforts should not focus on attempting to decrease length of culture.

The parameters included in this analysis are not exhaustive as additional factors are known to influence production COG in bioprocesses, such as batch failure rate, recovery yield of unit operations, or different wages (40, 59, 60). Parameter selection was based on reported impact of COG bioprocesses for allogeneic cell therapy (40, 41). Cell yield is usually a very significant cost driver. In the case of iNeut, although required cell numbers per dose will not be known until escalation-dose clinical trials, the literature on donor neutrophil transfusions suggests that at least 2 × 10^10^ cells would be necessary per transfusion in a prophylactic setting with transfusions every second day (1, 26). Although our analysis identifies larger batches of 104 L, equivalent to 10 prophylactic doses, being the most cost-efficient for a single bioreactor, this scenario assumes appropriate storage conditions are in place and minimal cell loss occurs post-thawing.

The impact of cell loss upon cryopreservation on iNeut COG can be indirectly studied in our model. In terms of impact on COG, a 30% cell loss post-cryopreservation is equivalent to a 30% decrease in production (Figure 5). Therefore, the critical impact of produced yield discussed is also relevant to cryopreservation induced cell loss. Based on this analysis, it will be critical to optimize a protocol to preserve iNeut while minimizing cell loss.

## Conclusions

Here we identified the most cost-effective production scenario for iNeut to be 104 L in a single Wave Bioreactor using the 27-day protocol. This translates into a production scale target for a scaled-out company aiming to produce iNeut to prevent neutropenia. Evidenced areas of opportunity for cost optimization included sourcing cheaper materials, solving the current obligate requirement for UCB, increasing cell yield and developing a successful cryopreservation protocol. It should be noted that downstream processes such as fill-and-finish or transport were not analyzed in the present work. Furthermore, labor contribution was maintained constant at all scales, while processing a 2 dose-batch may has less labor requirement than a 50-dose batch.

Although iNeut remain to be clinically tested, a positive outcome may qualify this therapy to be designated as regenerative medicine advanced therapy (RMAT) by relevant authorities (61, 62). Analyzing in advance bioprocess economics and areas of opportunity are critical to support commercial success. This research acts as an exemplar study for allogenic cell-based therapies, and can be used to inform development of other emerging cell therapies developed in suspension culture technologies.

## Author Contributions

MT-A developed the model, performed *in silico* experiments, analyzed results, wrote and edited the manuscript. RH and EC participated in study design, provided data, analyzed results, wrote and edited the manuscript. MR-P reviewed the model and edited the manuscript. MB conceived the study, interpreted results, wrote and edited the manuscript.

### Conflict of Interest Statement

The authors declare that the research was conducted in the absence of any commercial or financial relationships that could be construed as a potential conflict of interest.

## Footnotes

^1^*Sartorius Group Scalable Single-Use Centrifugation Systems*. Available online at: https://www.sartorius.com/sartorius/en/EUR/ksep-systems (Accessed December 4, 2017).
