# Supplementary material for: Ex vivo Manufactured Neutrophils for Treatment of Neutropenia—A Process Economic Evaluation
Source: Front Med (Lausanne). 2019 Mar 1;6:21. doi: 10.3389/fmed.2019.00021 (PMC6405517; doi:10.3389/fmed.2019.00021)
Supplement: Supplementary file 1 [file Table_1.DOCX]

**Supplementary Table 1.** Cost data used to populate the Biosolve model for neutrophil production. Costs that were cell number dependent were consequently adjusted.

|  | **Cost Component** | **Cost (US $)** | **Supplier** |
| --- | --- | --- | --- |
| **Equipment** | CliniMACS | $46,678 | Miltenyi Biotec |
|  | kSep | $200,000 | Sartorius Stedim |
|  | Wave Bioreactor | Varies depending on the production scale according to the next equation:  *Bioreactor cost [US $] = 18835(Bioreactor volume desired)^0.41^* | GE Healthcare |
| **Materials**  *Costs include discount for bulk purchase*  *(30%/90%)* | StemLine™ II, *GMP grade* | $550.2/$78.6 p*er 3L* | Sigma-Aldrich |
|  | Stem Cell Factor, *GMP grade* | $1,120/$160 per 50μg | Sigma-Aldrich |
|  | Granulocyte Colony Stimulating Factor, *GMP grade* | $520.1/$74.3 per 25μg | Sigma-Aldrich |
|  | Thrombopoietin Peptide Mimetic, *non-GMP* | $700/$100 per 50mg | CanPeptide |
| **Consumables**  *No discount considered* | CliniMACS consumables and reagents | $4,000/10 blood cord units (equivalent to a maximum of 50 million CD34^+^ cells collected) | Miltenyi Biotec |
|  | Wave Bioreactor Bags | The cost changes according to the scale being analyzed. Two bags were used per batch. The total cost for bags was calculated with the following equation:  ***Bioreactor Bag*** ***1*** *[US $] = 5.35*$\times$ *(Volume of bioreactors used [L])] + 242.73*  ***Bioreactor Bag 2*** *[US $] = (1/10)*$\times$ *(Cost of Bioreactor Bag 1)* | GE Healthcare |
|  | kSep consumables | $2,500 per 30 billion cells | Sartorius Stedim |
| **Quality Control** | Quality Control Tests, per batch | $4650 (Heinzle et al., 2006) | Variable |
