# Supplementary material for: Ex vivo Manufactured Neutrophils for Treatment of Neutropenia—A Process Economic Evaluation
Source: Front Med (Lausanne). 2019 Mar 1;6:21. doi: 10.3389/fmed.2019.00021 (PMC6405517; doi:10.3389/fmed.2019.00021)
Supplement: Supplementary file 2 [file Table_2.DOCX]

**Supplementary Table 2.** Culture volumes corresponding to best improvement scenarios (80% and 90%) using the 15-day or 27-day protocols and either 30% or 90% material cost supplier discount.

|  |  | Culture scale (L) providing % improvement over cheapest COG | |
| --- | --- | --- | --- |
|  | **Bulk material discount** | **80%** | **90%** |
| 15-day protocol | 30% discount | 57.5 | 104 |
|  | 90% discount | 58.5 | 106.5 |
| 27-day protocol | 30% discount | 57.5 | 104 |
|  | 90% discount | 58 | 105.5 |
| **Average** |  | **57.875** | **105** |
